# Management of necrotizing pneumonia with bronchopleural fistula caused by multidrug‐resistant *Acinetobacter baumannii*

**DOI:** 10.1002/rcr2.662

**Published:** 2020-09-15

**Authors:** Allen Widysanto, Maranatha Liem, Karina Dian Puspita, Cindy Meidy Leony Pradhana

**Affiliations:** ^1^ Department of Respiratory Medicine Siloam Hospital Lippo Village Tangerang Indonesia; ^2^ Faculty of Medicine Pelita Harapan University Tangerang Indonesia

**Keywords:** *Acinetobacter baumannii*, bronchopleural fistula, hydropneumothorax, necrotizing pneumonia

## Abstract

We report the case of a 53‐year‐old male that presented to our hospital with a history of a brain tumour. He was hospitalized 10 days prior in another hospital. Before surgery, he complained of mild cough. Routine chest radiography demonstrated right upper lobe consolidation which was diagnosed as hospital‐acquired pneumonia. Broad‐spectrum empirical antimicrobial was initiated. After surgery, his clinical condition deteriorated and he felt breathlessness. Chest radiography and computed tomography (CT) scan without contrast revealed necrotizing and cavitating pneumonia complicated by bronchopleural fistula (BPF) and hydropneumothorax. Sputum culture revealed infection of multidrug‐resistant *Acinetobacter baumannii* (MDRAB). Despite optimal antibiotic therapy, BPF and hydropneumothorax failed to resolve and surgical approach was performed to debride the necrotic area and seal the fistula. After a month in the hospital, he was discharged and the serial chest X‐ray showed good recovery of the lung.

## Graphical abstract

We present a case of necrotizing pneumonia (NP) caused by multidrug‐resistant *Acinetobacter baumannii* that also complicated by hydropneumothorax and bronchopleural fistula (BPF). Despite optimal antibiotic therapy, BPF and necrotic tissue often fail to resolve and surgical debridement or resection is required. This report highlights our approach and management for patients with severe NP caused by multidrug‐resistant microorganisms including timing to perform surgery and choice of antimicrobial treatment.

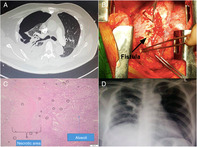

## Introduction

Necrotizing pneumonia (NP) is a severe form and potentially fatal complication of pneumonia that is characterized by progressive lung necrosis which may lead to pulmonary gangrene. The disease causes destruction of underlying lung parenchyma resulting in multiple small, thin‐walled cavities that involve patchy, segmental, lobar, or even the entire lung [1, 2].

We present a case of NP caused by multidrug‐resistant *Acinetobacter baumannii* (MDRAB) that also complicated by hydropneumothorax and bronchopleural fistula (BPF). Despite optimal antibiotic therapy, BPF and necrotic tissue often fail to resolve and surgical debridement or resection is required. This report highlights our approach and management for patients with severe NP caused by multidrug‐resistant microorganisms including timing to perform surgery and choice of antimicrobial treatment.

## Case Report

**Figure 1:**
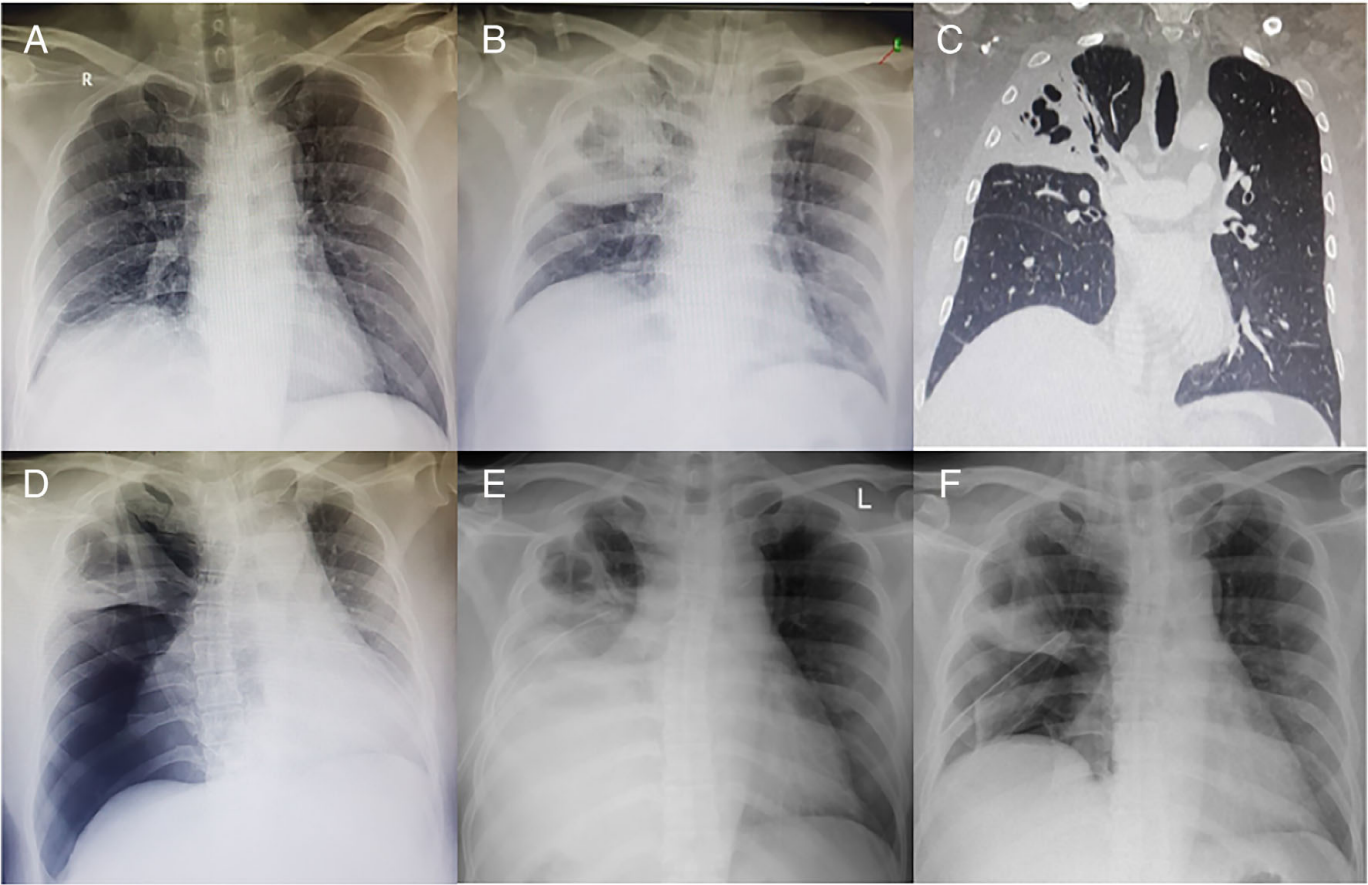
(A) Chest X‐ray that was obtained a week before the patient came to our hospital. (B) At our hospital, pre‐surgical chest X‐ray showing round shape consolidation with air space inside the right upper lung. (C) Coronal section of non‐contrast computed tomography (CT) scan showing consolidation with air bronchogram and multiple coalescence cavitary opacities at right upper lung. (D) Prior to the chest tube insertion (three days after starting tigecycline and moxifloxacin), chest X‐ray showing large pneumothorax and collapse of the right lung with contralateral mediastinal shift. (E) A day after chest tube insertion, chest X‐ray demonstrated change in air–fluid level caused by hydropneumothorax. (F) Two weeks after the chest tube insertion, chest X‐ray showing that the inflammation resolved but the lung failed to expand and the pneumothorax persists.

A 53‐year‐old non‐smoker man with history of type 2 diabetes mellitus and a brain tumour was referred to our hospital for tumour removal surgery. He was hospitalized 10 days prior in another hospital. Initial chest X‐ray showed consolidation with air space inside the right upper lobe indicating pneumonia with possibly abscess or cavitary formation (Fig. 1B). Meropenem and amikacin were initiated empirically according to our local antibiotic resistant profile before the surgery.

A day after the surgery he started to complain of breathlessness. Vital signs were notable for a respiratory rate of 28 breaths per minute on room air with saturation of 95%, heart rate of 103 beats per minute, and blood pressure 100/60 mmHg. On examination, he was alert, perfusion was normal, and on auscultation there were diminished breath sounds at his right upper chest. Laboratory findings demonstrated a leucocyte count of 22.17 per μL, haemoglobin of 13.6 g/dL, and platelet count of 287 per μL. Procalcitonin level was 0.33 ng/mL with erythrocyte sedimentation rate of 67 mm/h. Screening for HIV, autoimmune disease, tuberculosis, and fungal infection was negative. A computed tomography (CT) scan without contrast showed consolidation with multiple coalescing cavitary opacities of the right upper lung (Fig. 1C). Bronchoscopy was then performed and showed copious amount of thick mucoid secretion mainly from the right upper bronchus. Culture from tracheal aspiration sputum and bronchial lavage revealed growth of MDRAB susceptible only to tigecycline and resistant to penicillin group, ampicillin/sulbactam, cephalosporins, carbapenem group, and quinolone (levofloxacin and ciprofloxacin). Blood culture showed no growth of any microorganism. The antibiotic was then transitioned to tigecycline and moxifloxacin. Later, fluconazole and metronidazole were also added to cover other possible fungi and anaerobe.

**Figure 2:**
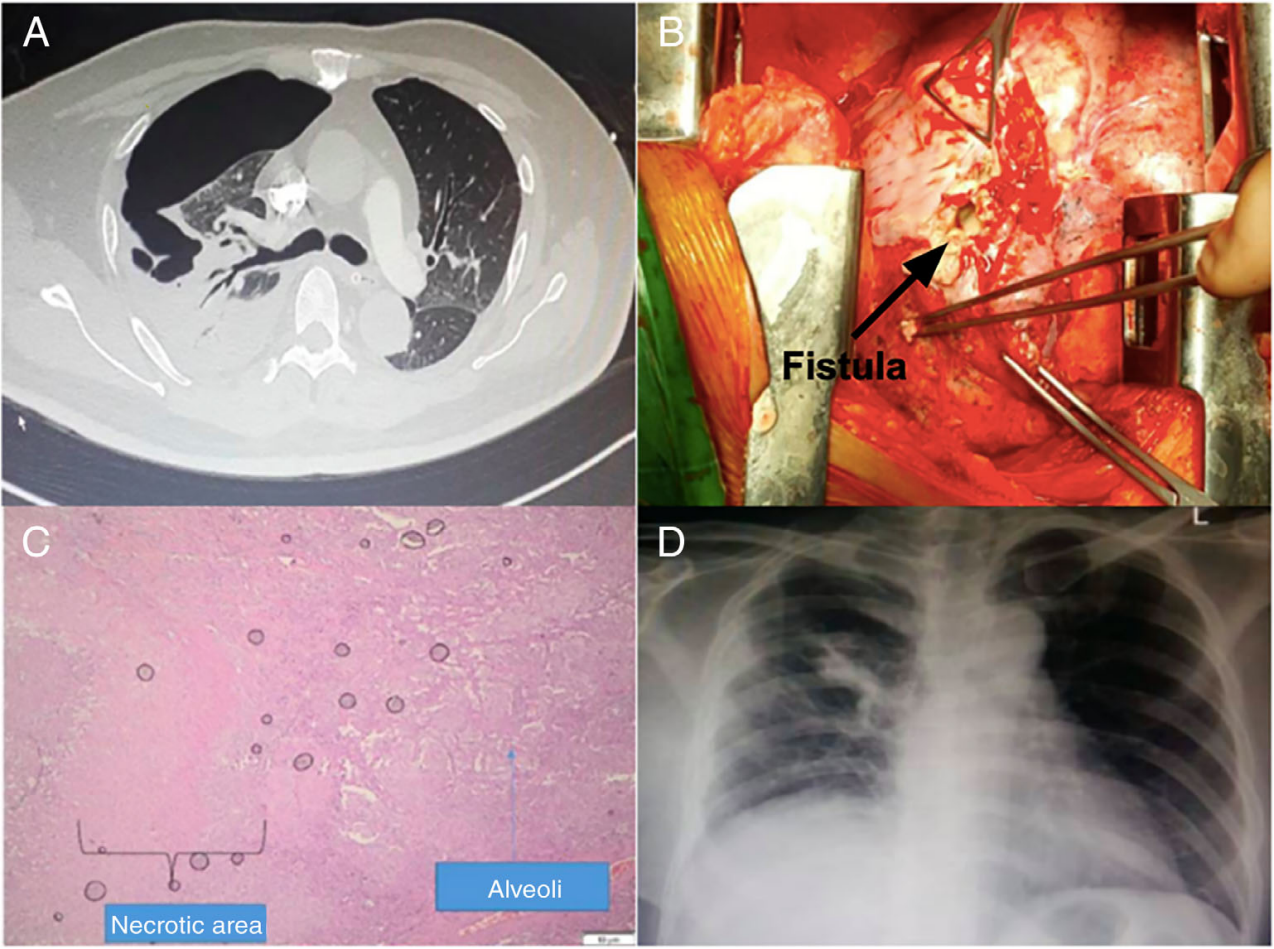
(A) Axial section of non‐contrast computed tomography (CT) scan showing right upper lung collapse with large fistula and pneumothorax. (B) Surgical image showing large fistula surrounded by pus and necrotic tissue at the right upper lung. (C) Histopathology showing necrotic area surrounding lung alveolus. (D) Chest X‐ray that was obtained a day before the patient was discharged showing the right rung has expanded and no sign of pneumothorax.

Three days later, he complained of pain at his right chest and dyspnoea was getting worse. His respiratory rate increased and saturation decreased to 91% at 2 L oxygen. Chest X‐ray (Fig. 1D) and CT scan (Fig. 2A) were repeated and showed consolidation with air bronchograms, large BPF, atelectasis, and collapse of the upper right lung. A chest tube was placed to allow drainage of serous fluid and air which indicated hydropneumothorax (Fig. 1E). After two weeks of chest tube insertion, chest X‐ray showed decreased inflammation and air–fluid level, but the collapsed lung failed to expand (Fig. 1F). Following this failure, the decision to perform surgery was made. The surgery was delayed for one week to allow recovery of the lung from the infection and the inflammation to achieve clinically stable condition. After 15 days administration of tigecycline and moxifloxacin, repeated sputum culture showed no growth of any microorganism. Following the improvement of his clinical condition and all of the inflammatory markers, the antibiotic was de‐escalated a week before surgery to oral amoxicillin‐clavulanate to avoid risk of antimicrobial resistance. Cefoperazone‐sulbactam was added as a prophylactic before surgery. Open thoracotomy surgery was then performed after 25 days of starting susceptible antibiotic to evacuate part of the necrotic lung and seal the fistula. Exploration showed a large fistula surrounded by pus necrotic tissue at the right upper lung (Fig. 2B). The fistula was sealed followed by removal of the purulent exudate and necrotic portion of the upper right lung.

The next day, chest X‐ray demonstrated atelectasis of his right lung. Continuous positive airway pressure with low positive end‐expiratory pressure (PEEP) was administrated to re‐expand the lung. After six days, the patient was able to mobilize. Pus culture showed no growth of any microorganism. Surgical pathology revealed necrotic lung tissue surrounded by inflammatory cells (Fig. 2C). After five days, the lung expanded completely (Fig. 2D) and no residual air leak was found and the chest drain was removed. He recovered very well and was discharged.

## Discussion

Our patient developed NP from nosocomial infection. *Acinetobacter baumannii* is a rare cause of NP. It is often associated with immunocompromised and hospitalized patients. It also exhibits relatively high virulence and antimicrobial resistance compared with other organisms [1]. He has diabetes as the comorbidity and had previously hospitalized at another hospital for one week. His clinical condition rapidly deteriorated after tumour removal surgery possibly due to resistance to empiric antimicrobials. Catha et al. conducted review of five NP patients and found that NP showed rapid clinical deterioration in all of the patients [3]. Most patients with MDRAB infection show rapid clinical deterioration and usually do not respond to the first‐line antibiotic treatment. MDRAB was not considered as the cause of infection until the culture tests were done.

Hydropneumothorax is the most commonly reported complication of NP with BPF. Necrosis and breakdown of the lung parenchyma allow destruction of bronchial wall and communication between lung parenchyma and pleural space. The presenting signs and symptoms include cough, progressive dyspnoea, chest pain, absence of breath sounds, hyperresonance of chest percussion, and signs and symptoms of tension pneumothorax. New or increasing air–fluid level is often seen on chest X‐ray. Diagnosis of BPF can be confirmed by chest CT scan which reveals free air–fluid levels within the pleural space with fistulous communication. Adequate pleural drainage needs to be placed. Medical management should include drainage and reduction of the pleural space, antibiotics, nutritional supplementation, and adequate ventilator management if ventilated. After one to three weeks, surgical management is an option for BPF in large tumours (>10 mm) that complicated parenchymal disease such as NP [4].

There are no clear consensus when to perform surgery in NP patient. The indication to perform surgery in our patient was persistent BPF. However, there are no guidelines stating optimal timing to perform surgery in NP patients. Reimel et al. suggest waiting until patients have been clinically stable, which favour for better surgical outcomes. As the infection and inflammation progress, necrotic areas coalescence to form large cavities and pus collections surrounding the area make them under perfused. Delayed surgery allows “areas of lung that had documented perfusion to resolve, further localizing the area that actually required resection” [2]. Chatha et al. report that delaying surgery increases the risk of sepsis, multiple organ failure, and bacterial contamination of contralateral lung [3].

Antibiotics were administrated based on the clinical condition, microorganism culture, and inflammation markers such as C‐reactive protein (CRP) and procalcitonin level. Administration of multiple antimicrobial agents that cover all microorganisms including fungal and anaerobic microorganism is very important. It is necessary to change the antimicrobial therapy based on not only the microbial culture, but also the clinical status, laboratory results, and imaging. Tigecycline is considered as a choice in MDRAB. From the multivariate analysis that was conducted by Zhou et al. in 77 patients with MDRAB, treatment with tigecycline showed clinical success and reduction of 30‐day mortality rate when the minimum inhibitory concentration (MIC) < 2 mg/dL [5].

Surgery is aimed to resect and debride the necrotic tissue, then seal the fistula to allow expansion, and protect healthy lung tissue. Clinicians must be aware of post‐operative complications caused by augmentation of systemic inflammatory response triggered by surgery [2]. Chatha et al. also recommended open thoracotomy approach to optimizing the resection and distinguishing viable and non‐viable lungs [3].

From this case, we learn that clinicians need to be aware of severe complicated pneumonia that can be caused by unusual pathogen like MDRAB, which does not respond to standard antimicrobial therapy. Perfect timing to perform surgery by considering the pathophysiology and progression of the disease is important in managing NPs with complication like BPF that does not respond to standard therapy.

### Disclosure Statement

Appropriate written informed consent was obtained for publication of this case report and accompanying images.
